# Supplementary material for: Platform-directed allostery and quaternary structure dynamics of SAMHD1 catalysis
Source: Nat Commun. 2024 May 6;15:3775. doi: 10.1038/s41467-024-48237-w (PMC11074143; doi:10.1038/s41467-024-48237-w)
Supplement: Supplementary file 1 — Supplementary Information [file 41467_2024_48237_MOESM1_ESM.pdf]

Supplementary Information

Platform-directed allostery and quaternary structure dynamics of SAMHD1 catalysis

Oliver J. Acton, Devon M. Sheppard, Simone Kunzelmann, Sarah J. Caswell, Andrea Nans, Ailidh J. O. Burgess, Geoff Kelly, Elizabeth R. Morris, Peter B. Rosenthal and Ian A. Taylor

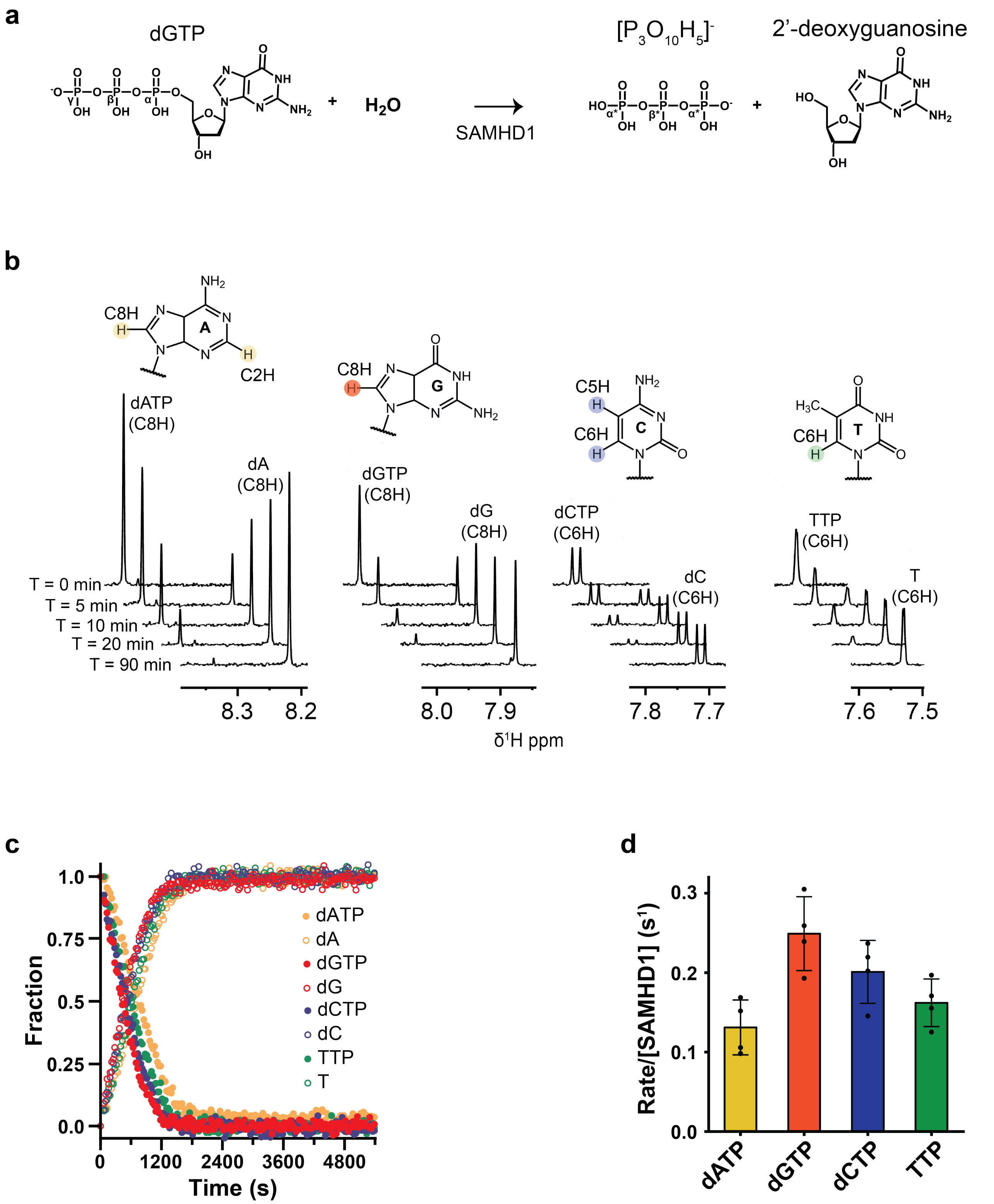

Supplementary Fig. 1. | ^{1}H NMR analysis of SAMHD1 deoxynucleotide hydrolysis. (a) Chemical reaction scheme of the SAMHD1 enzymatic reaction. A dGTP substrate is hydrolysed by SAMHD1 to produce deoxyguanosine and triphosphate products. In the

chemical structures, the α, β and γ-phosphate of dGTP together with the α*- and β*-phosphate of triphosphate are labelled. (**b**) Time course ^{1}H NMR data recorded for a SAMHD1 hydrolysis reactions containing 2 μM SAMHD1, 0.2 mM GTP AL1-activator and 0.5 mM of deoxynucleotides dATP, dGTP, dCTP and TTP. Peak resonances from each deoxynucleoside are shown at the times of reaction indicated. (**c**) Fraction of each dNTP substrate and dN product present through the course of the hydrolysis reaction. (**d**) Initial rates of hydrolysis of each dNTP were determined from slopes derived from the data measured in the linear phase of the reaction shown in **c**. Bar height represents the mean of the plotted data points and error bars represent the s. d. of the mean of four independent measurements. Source data for **c & d** are provided in the Source Data file.

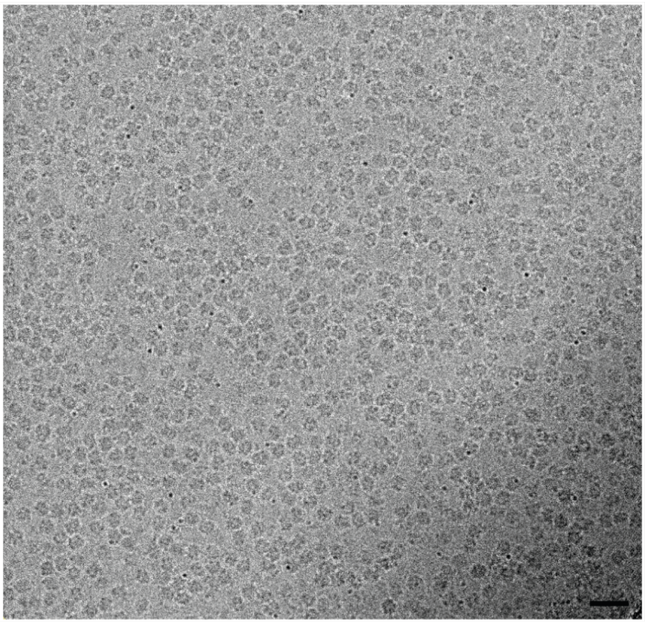

Supplementary Fig. 2. | CryoEM Data collection. (a) Single micrograph of SAMHD1 particles from a Krios dataset collected at 300 kV, nominal magnification of 130000, (1.08Å/pix), defocus ~ -2.0µm (Scale bar = 20 nm).

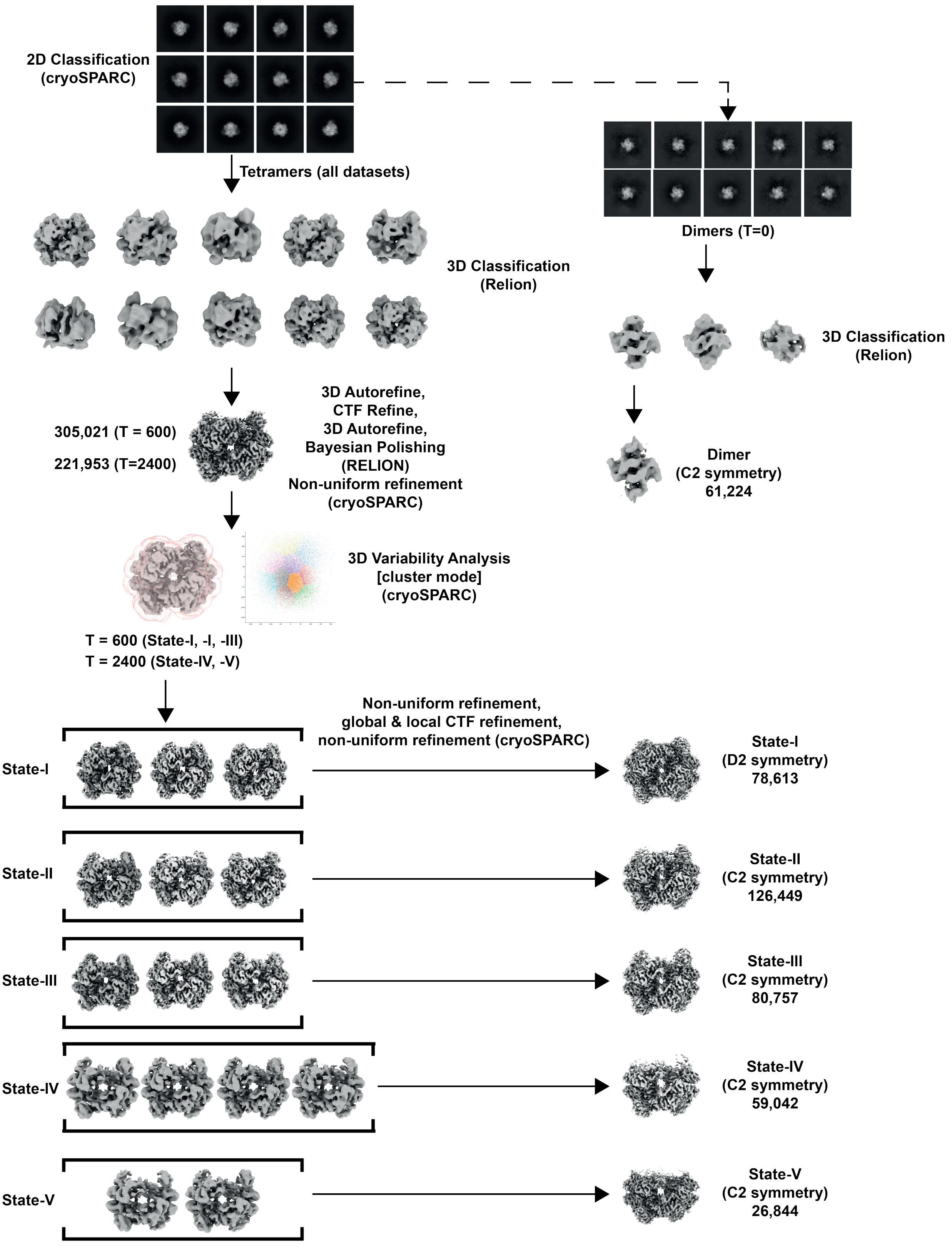

Supplementary Fig. 3. | CryoEM image processing. Overview of the image processing and refinement procedures used to produce maps and determine SAMHD1 State I-V structures. The scheme is representative of the State-I, -II and -III T = 600 s dataset and

State-IV and -V T = 2400 s dataset processing procedures. Particle counts for the consensus map and following 3D variability analysis are given for each map. The processing of the Dimer from the T = 0 s timepoint showed only low resolution highly similar maps after 3D classification and is shown in a parallel workflow. The software packages used at each stage are indicated and only unique steps are shown for each refinement scheme.

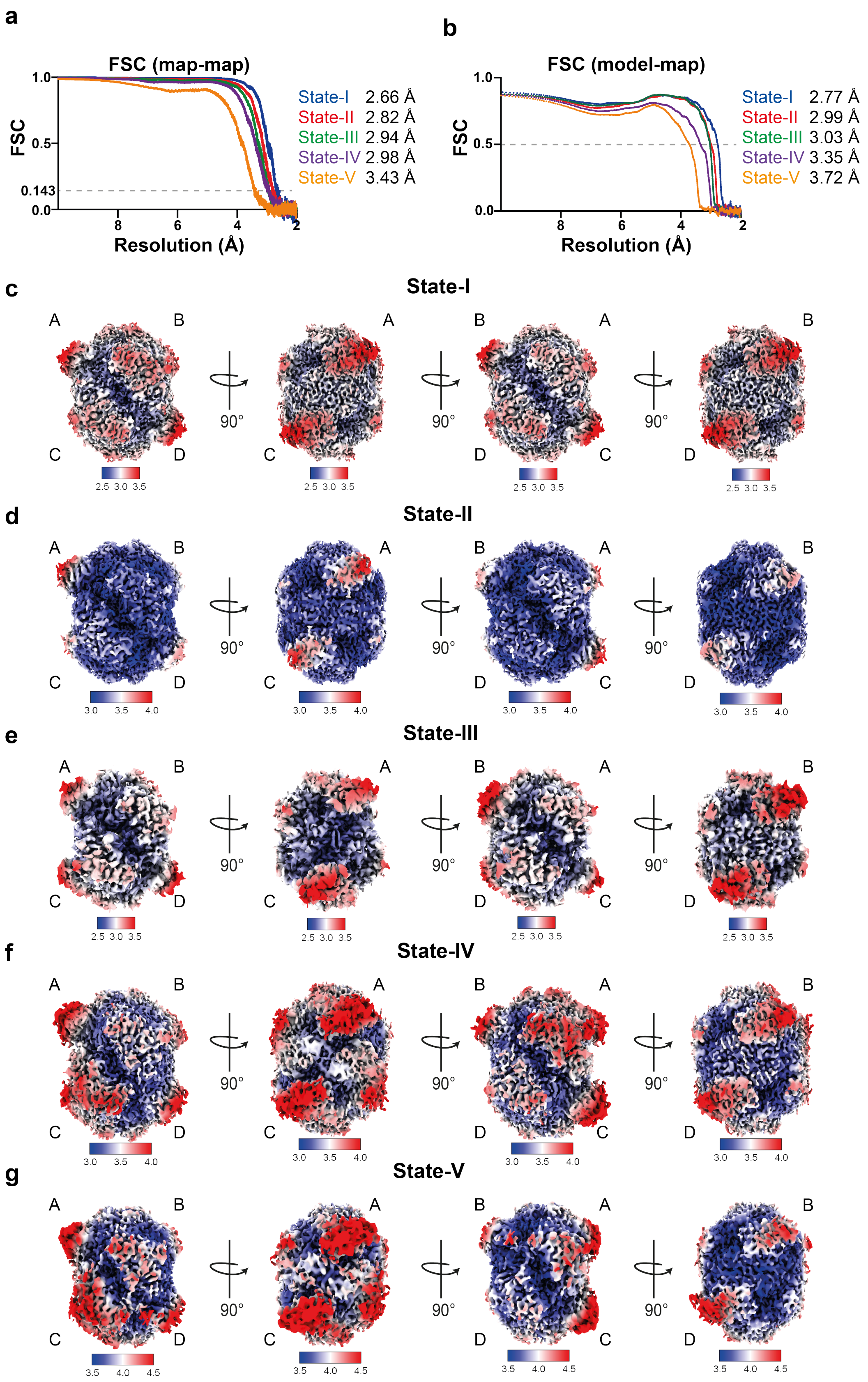

Supplementary Fig. 4. | SAMHD1 States-I to -V reconstruction and resolution (**a**) Half-map-map and (**b**) map-model FSCs, for State-I, State-II, State-III, State-IV, and State V

particles. The half-map resolution cut-offs at FSC of 0.143 are State-I 2.66 Å, State-II 2.82 Å, State-III 2.94 Å, State-IV 2.98 Å and State-V 3.43 Å. The model vs map FSC calculated from final model refinement in PHENIX have resolution cut offs at FSC of 0.5 of; State-I 2.77 Å, State-II 2.99 Å, State-III 3.03 Å, State-IV 3.35 Å and State-V 3.72 Å. (**c - g**) Views of the electron potential maps contoured at 3σ, looking down the two-fold symmetry axes of the (**c**) State-I D2 particle, (**d)** State-II C2 particle, (**e**) State-III C2 particle, (**f**) State IV C2 particle and (**g**) State-V C2 particle. In each panel the positions of the A, B, C and D monomers are indicated, colouring is by local resolution (Å) determined in Relion 3.1 and according to the scale shown beneath.

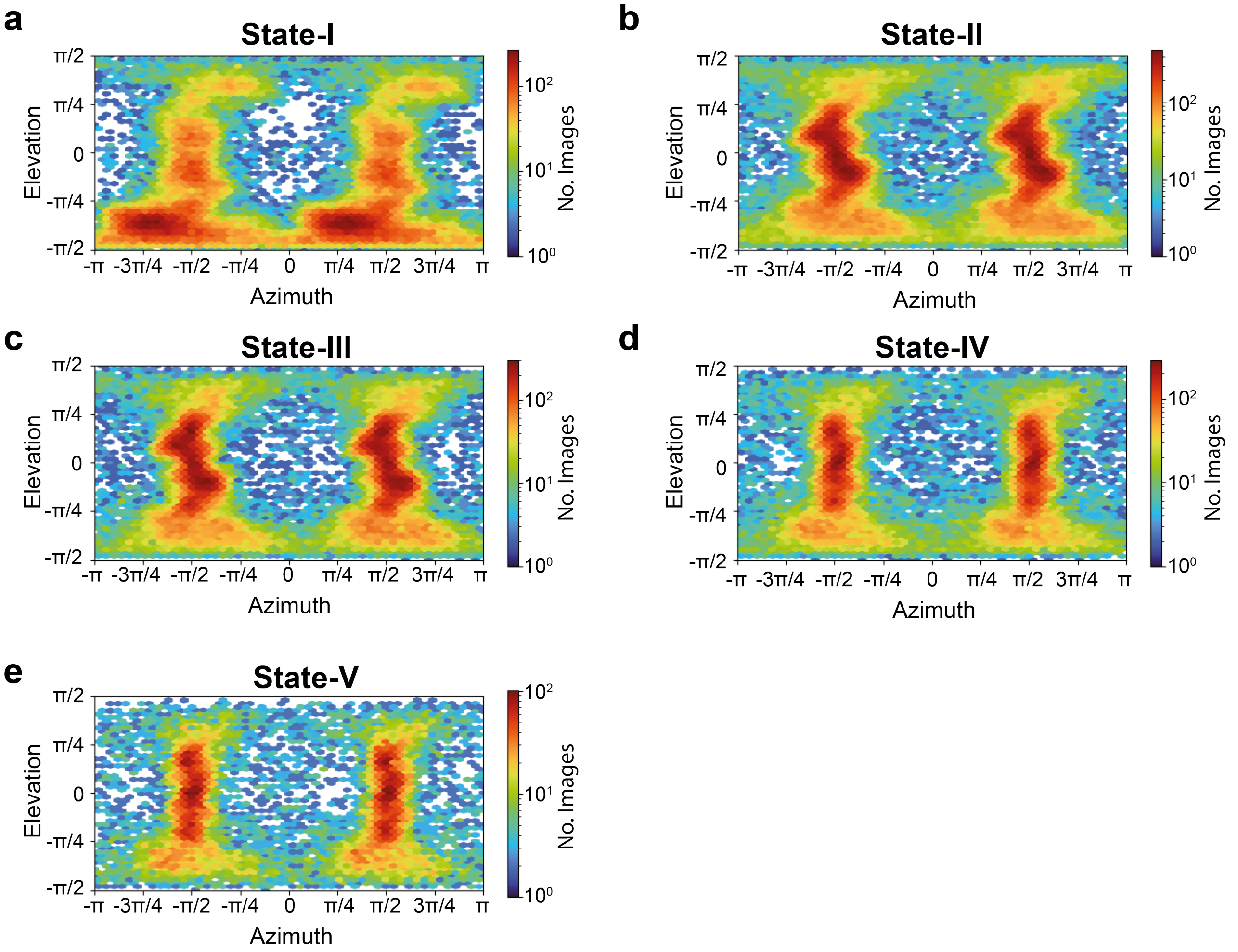

Supplementary Fig. 5. | Euler plots. Particle distribution plots calculate in after final refinement for (**a**) State-I, (**b**) State-II, (**c**) State-III, (**d**) State-IV and (**e**) State-V. Colouring is according to the No. of images scale on the right.

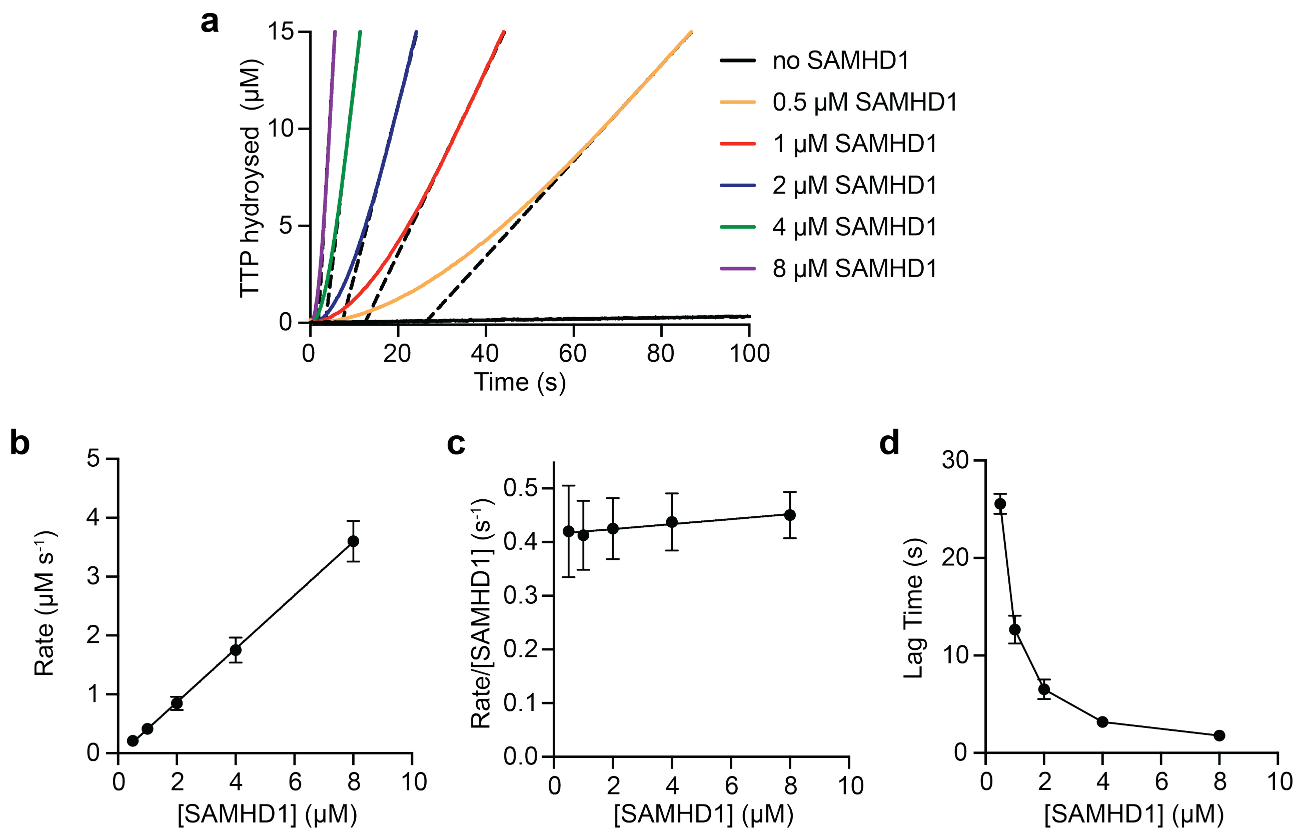

Supplementary Fig. 6. | Kinetic analysis of SAMHD1 lag phase. (**a**) Continuous SAMHD1-Ppx MDCC-PBP coupled assay measured in a stopped-flow apparatus. The MDCC-PBP fluorescence readout reports on phosphate release from the SAMHD1-Ppx coupled TTP hydrolysis. Time courses of TTP hydrolysis show a pronounced lag phase that precedes the linear steady-state phase and increases with decreasing SAMHD1 concentration. Dashed lines are best linear fits to each curve to give the time of the initial lag (x intercept) and the maximal hydrolysis rate after the lag phase (slope). (**b**) Plot of the rate of TTP hydrolysis vs SAMHD1 concentration. The data is taken from the maximum slopes derived from the data in **a**. The hydrolysis rate has a linear dependence on SAMHD1 concentration with k_{cat} = 0.4 s^{-1}. (**c**) Plot of the data in **b** as enzyme normalised rate k_{cat} against SAMHD1 concentration. The data indicates that after the initial lag phase the rate is first order with respect to SAMHD1 concentration. (**d**) Plot of lag time against SAMHD1 concentration, data taken from the x-axis intercepts of the linear fits of the data in **b**. The decrease of lag time with higher SAMHD1 concentration indicates that the lag is caused by

the slow assembly of SAMHD1 catalytically active tetramers. In **b**, **c** and **d**, values are the mean and error bars represent the s. d. of the mean from three independent measurements. Source data for **a-d** are provided in the Source Data file.

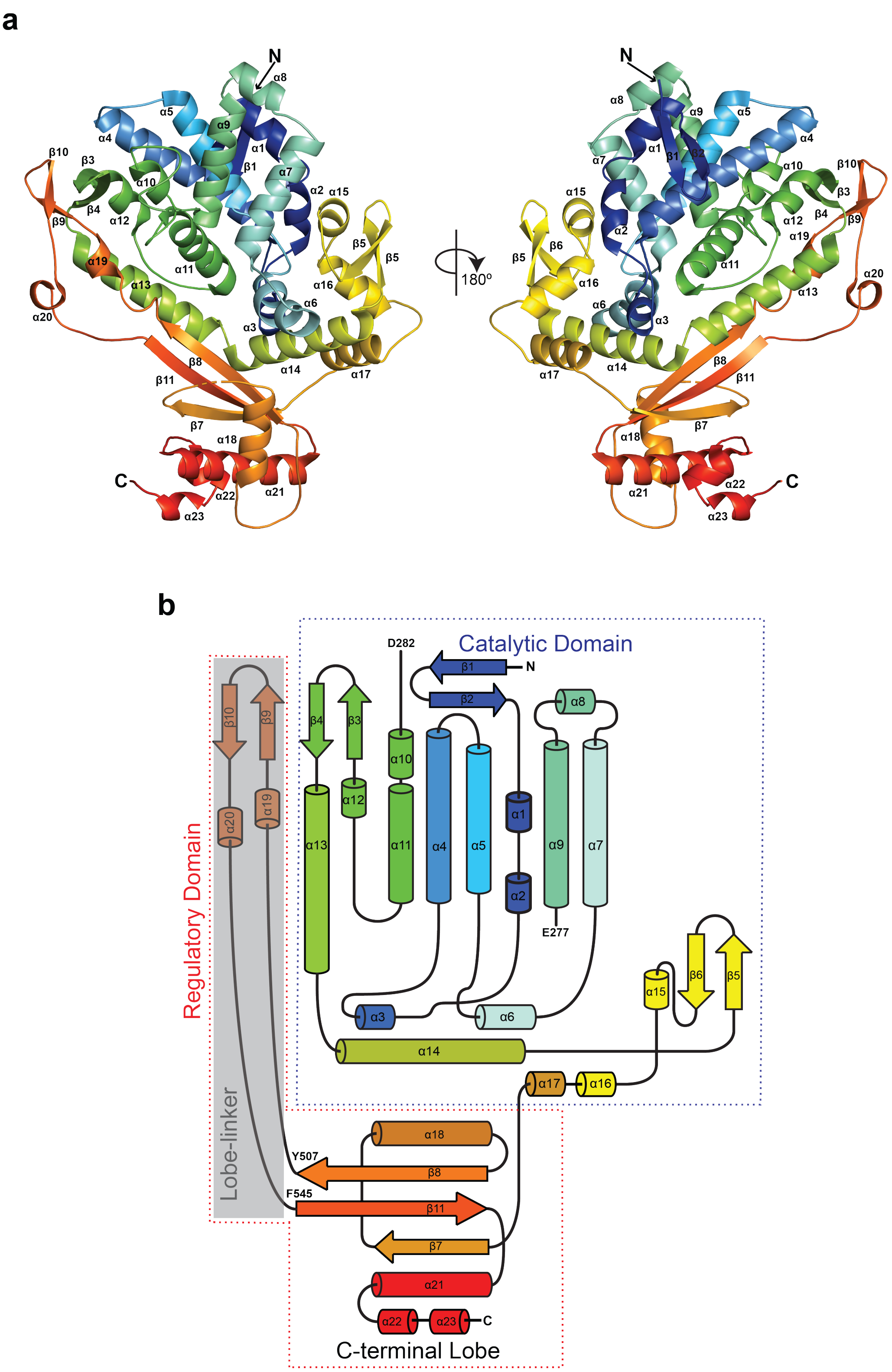

Supplementary Fig. 7. | SAMHD1 Secondary structure and topology. (a) Front and rear view of the SAMHD1 HD-monomer shown in ribbons representation coloured by spectrum (blue to red) from the N- to C-terminus. α-helices and β-strands are numbered sequentially from the N-terminus. (**b**) Topology diagram of SAMHD1 HD-monomer, secondary structure

elements are represented by arrows (β-strands) and cylinders (α-helices) that coloured sequentially from the N-terminus according to the scheme in **a**. The catalytic domain (residues D113-F454, β1 to α17) is bounded by the blue box. The regulatory domain (residues K455-N599, β7 to α23) that comprises the C-terminal lobe (residues K455-D506 & A546-N599) and the lobe-linker (residues Y507-F545) is bounded by the red box. Residues 277 - 282 constitute a disordered loop. The grey shading in the lobe linker region indicates the sequence that undergoes order-disorder-transition between SAMHD1 State-I to -V tetramers.

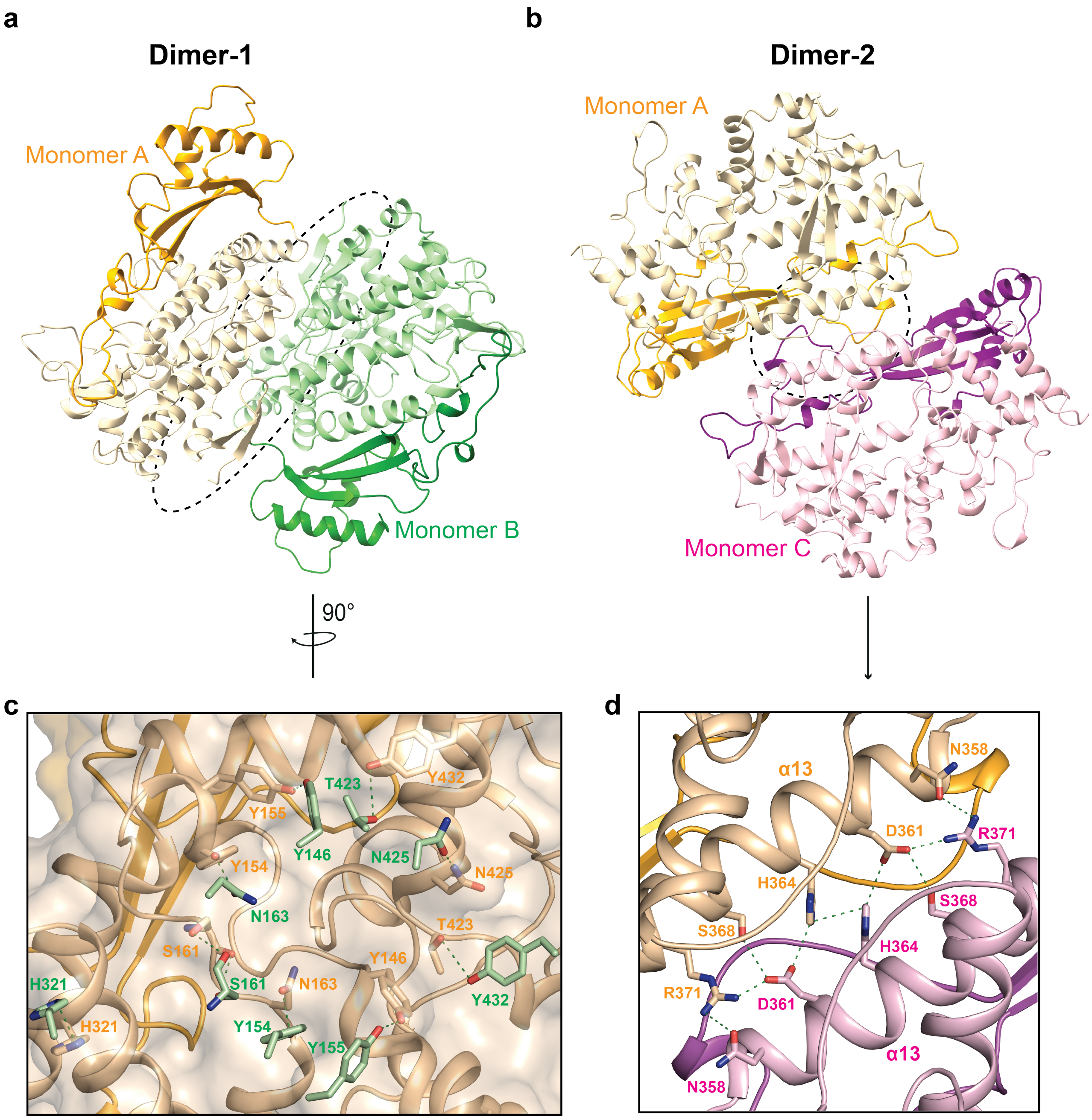

Supplementary Fig. 8. | SAMHD1 Dimer interfaces. The protein backbone shown in cartoon representation of (**a**) Dimer-1 and (**b**) Dimer-2 taken from a SAMHD1 tetramer - inhibitor complex crystal structure 6TX0 [https://www.rcsb.org/structure/6TX0] (**a**) Dimer-1 Monomer-A and Monomer-B catalytic and regulatory domains are shown in wheat and orange and pale green and green. (**b**) Dimer-2 Monomer-A and Monomer-C catalytic and regulatory domains are shown in wheat and orange and pale pink and magenta. The interaction surface of each dimer is indicated by the dashed elliptical boxes (**c**) Dimer-1

interactions, view is 90 ° from that in panel **a**, looking into the interface. Monomer A shown in semi-transparent surface with the backbone in cartoon. Side chains from Monomer-A and Monomer-B making interactions are shown in stick representation. Hydrogen bonding is represented by the dashed lines. (**d**) Dimer-2 interactions, view is perpendicular to the interface in the same orientation as panel **b**. Monomer-A and Monomer-C are shown in cartoon representation, centred on the packing of residues displayed on α13. Residues that make interactions are labelled and shown in stick representation. Hydrogen bond interactions are shown as dashed lines.

a

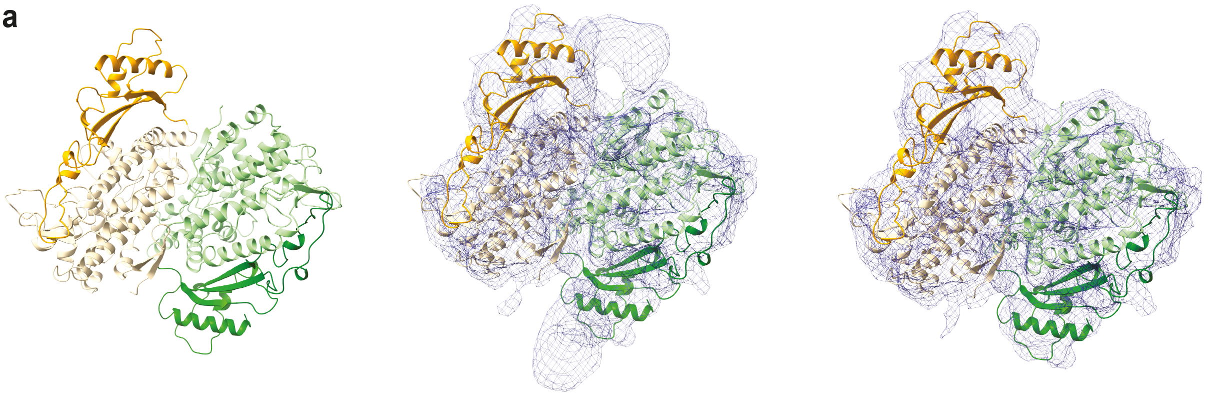

b

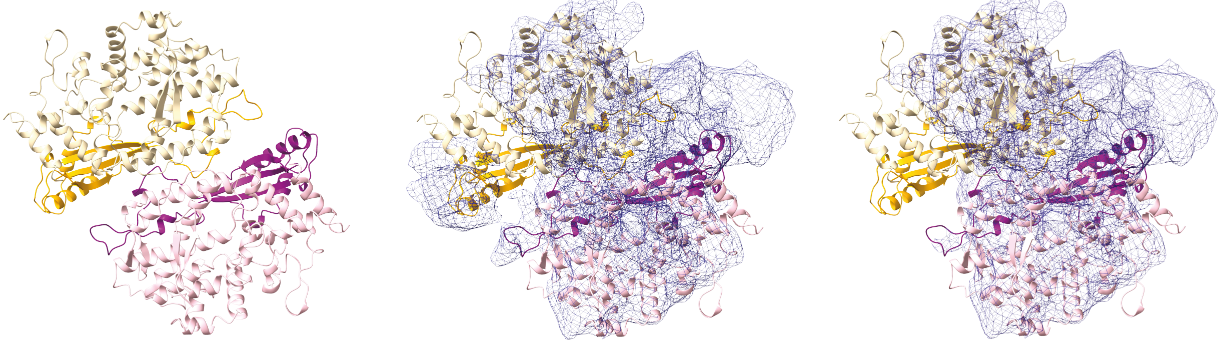

c

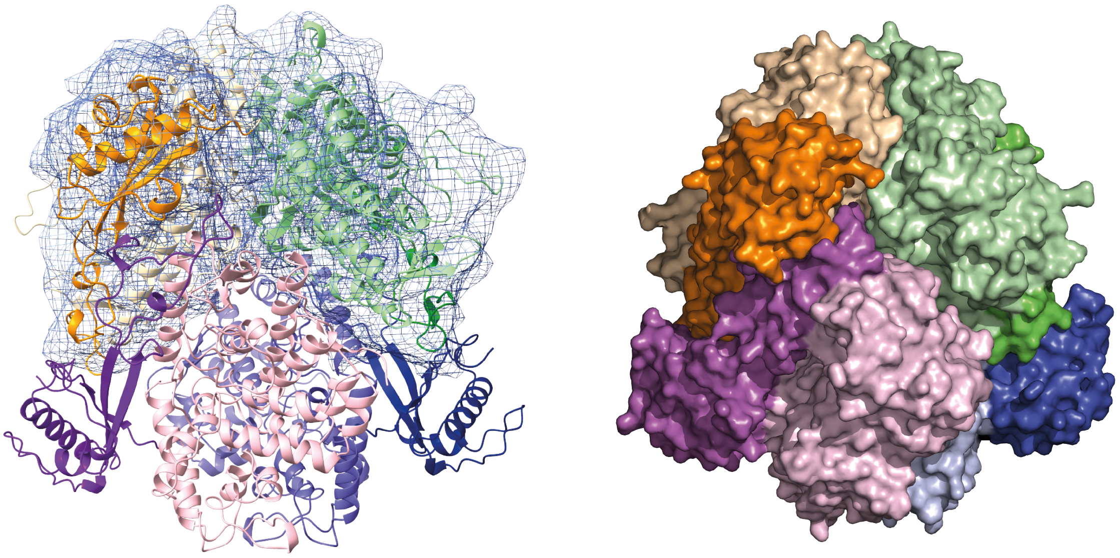

Supplementary Fig. 9. | SAMHD1 Dimers. (a-b) Rigid body fitting of SAMHD1 Dimer-pairs into the low resolution (13 Å) cEM map derived from imaging GTP-SAMHD1 particles. (**a**) (Left) Dimer-1, shown in cartoon representation, N-terminal catalytic and C-terminal regulatory domain are coloured wheat and orange (Monomer-A), pale green and green

(Monomer-B). (Middle) best rigid body fit of Dimer-1 into the cEM map, shown in blue/grey mesh. The weak density portions of the map that are not occupied are putatively assigned as the mobile SAM domains. (Right) Rigid body fitting of Dimer-1 into the cEM map but with the density for the putatively assigned SAM domains omitted. (**b**) (Left) Dimer-2, shown in cartoon representation, N-terminal catalytic and C-terminal regulatory domain are coloured wheat and orange (Monomer-A), pink and magenta (Monomer-C). (Middle) best rigid body fit of Dimer-2 into the cEM map. (Right) rigid body fitting of Dimer-2 into the SAM domain-omitted cEM map. Poor fitting of Dimer-2 compared with excellent fitting of Dimer-1 into the density confirm Dimer-1 as the stable solution dimeric species. (**c**) The State-I SAMHD1 D2 tetramer shown in cartoon representation (left) and surface representation (right). In both panels, the N-terminal catalytic domain, and C-terminal regulatory domain of the four monomers are coloured wheat and orange (Monomer-A), pale green and green (Monomer-B), pink and magenta (Monomer-C), pale blue and blue (Monomer-D). In the left-hand panel, the rigid body fitted cEM map derived from imaging GTP-SAMHD1 particles is superimposed onto the Monomer-A – Monomer-B Dimer-1 pair. The Dimer-2 pairs comprising Monomer-A – Monomer-C and Monomer-B – Monomer-D only form in the context of the assembled tetramer.

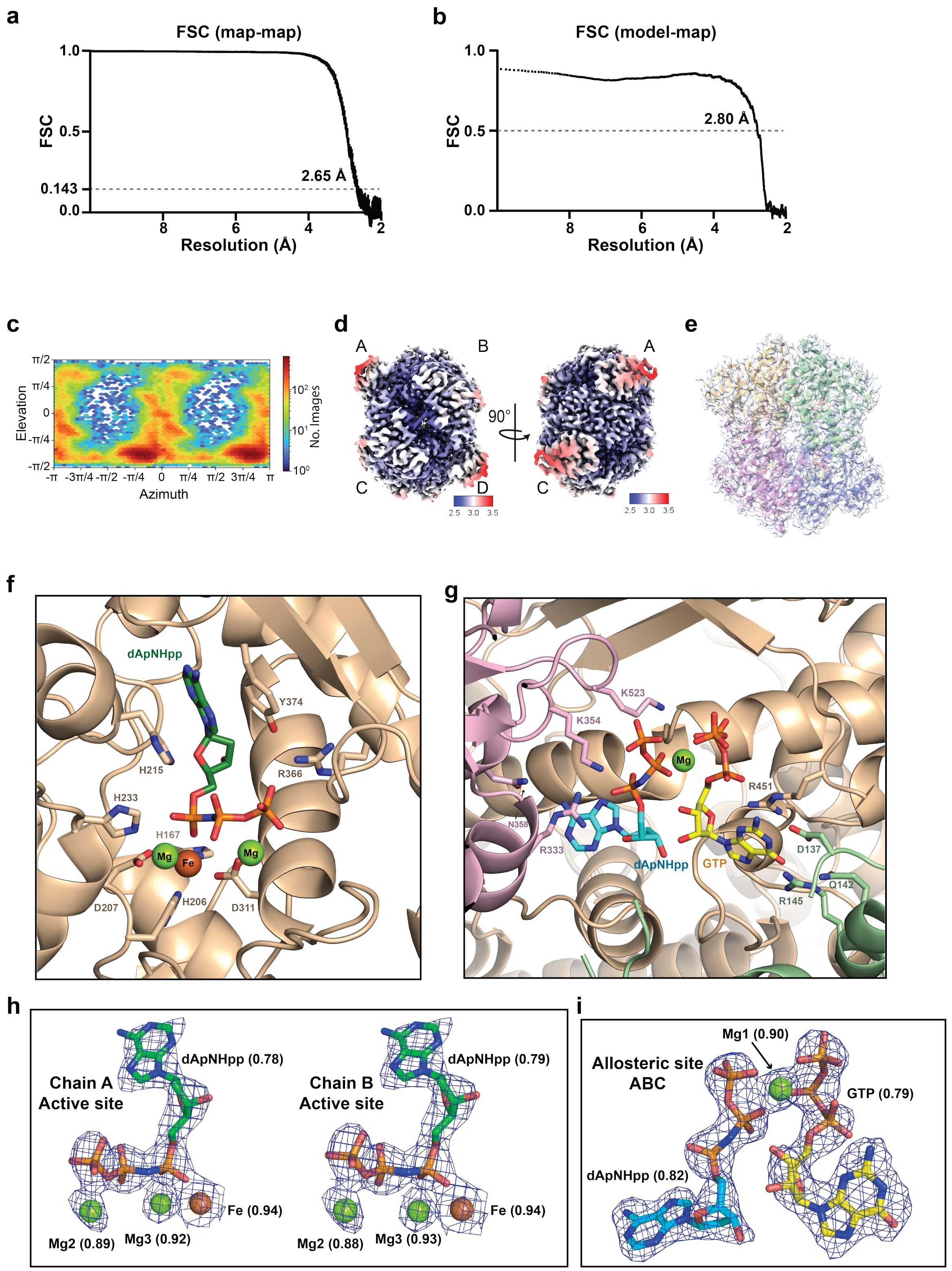

Supplementary Fig. 10. | SAMHD1 GTP-dApNHpp inhibitor structure. (**a**) Half-Map and (**b**) map-model FSC for the SAMHD1 GTP-dApNHpp structure. The half-map resolution cut-off at FSC of 0.143 is 2.65 Å. The map vs model FSC calculated from final model refinement in PHENIX has a resolution cut off FSC of 0.5 at 2.80 Å. (**c**) Particle distribution plot after

final refinement and D2 symmetry enforced. Colouring is according to the No. of images scale on the right. (**d**) Views of the electron potential map, contoured at 3σ and looking orthogonal (left) and down (right) the Dimer-2 two-fold symmetry axes of the SAMHD1 GTP-dApNHpp particle. The positions of the A, B, C and D monomers are indicated. Colouring is by local resolution (Å) determined in Relion 3.1 and according to the scale shown beneath. **(e)** The SAMHD1 GTP-dApNHpp D2 tetramer shown in cartoon representation fitted into the cEM map. The four monomers are coloured wheat (Monomer-A), green (Monomer-B), pink (Monomer-C) and blue (Monomer-D). (**f**) Active site of Monomer-A. The SAMHD1 protein backbone is shown in cartoon representation, coloured wheat. The active site-bound dApNHpp nucleotide and surrounding residues are shown as sticks with Fe and Mg metal ions shown as brown and green spheres, respectively. (**g**) Allosteric site ABC. The protein backbone is shown in cartoon representation; Monomer-A wheat, Monomer-B green and Monomer-C pink. GTP and dApNHpp nucleotides and surrounding residues are shown in stick representation. The Mg ion is shown as a green sphere. The conformation of the bound nucleotides and metal ions is identical to that observed in the high-resolution crystal structures of SAMHD1-dApNHpp complexes (6TX0 [https://www.rcsb.org/structure/6TX0]. (**h**) Electron density for dApNHpp, Fe and Mg metal ions bound at the active sites of Monomer-A and Monomer-B in the D2 symmetrised map. Representation is the same as panel **f** but rotated ~ 90 ° for clarity. Density (blue mesh) is contoured at 7.0 σ with map-model validation Q scores for nucleotides and ions displayed adjacently. (**i**) Electron density for GTP, dApNHpp and the bridging Mg ion at allosteric site ABC in the D2 symmetrised map. Representation is the same as panel **g**. Density is shown as a blue mesh, contoured at 8.0σ with map-model validation Q scores for nucleotides and ions displayed adjacently.

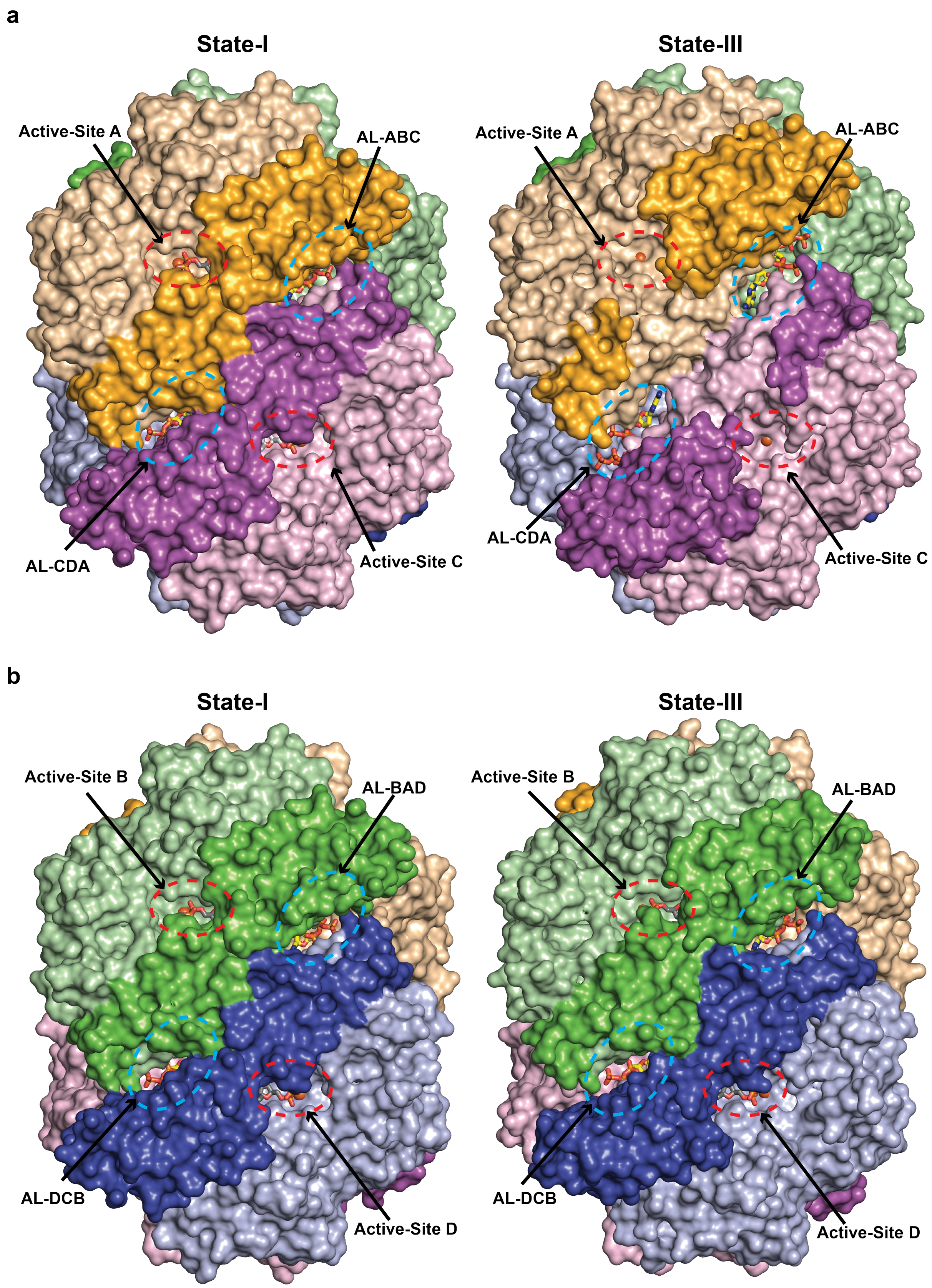

Supplementary Fig. 11. | State-I and State-III exposure of active and allosteric sites.

(**a & b**) (left) State-I and (right) State-III SAMHD1 tetramers viewed perpendicular to the AC

Dimer-2 (**a**) and to the BD Dimer-2 (**b**) twofold axes. Monomers are coloured as in Fig 1. The active and allosteric sites are indicated with arrows and circled in red (active) and in cyan (allosteric). In State-I regulatory domain lobe-linkers (orange, magenta, dark green and dark blue surface) occlude bound nucleotides in the active and allosteric sites. The State-I to State-III transition and resulting disordering of the regulatory lobe-linkers on the AC Dimer-2 face exposes active sites A and C and partially exposes the AL2-bound nucleotide at allosteric site ABC and CDA. The State-III BD Dimer-2 retains the tense state with no disordering of the lobe linkers and nucleotides remain occluded by the regulatory lobe-linkers.

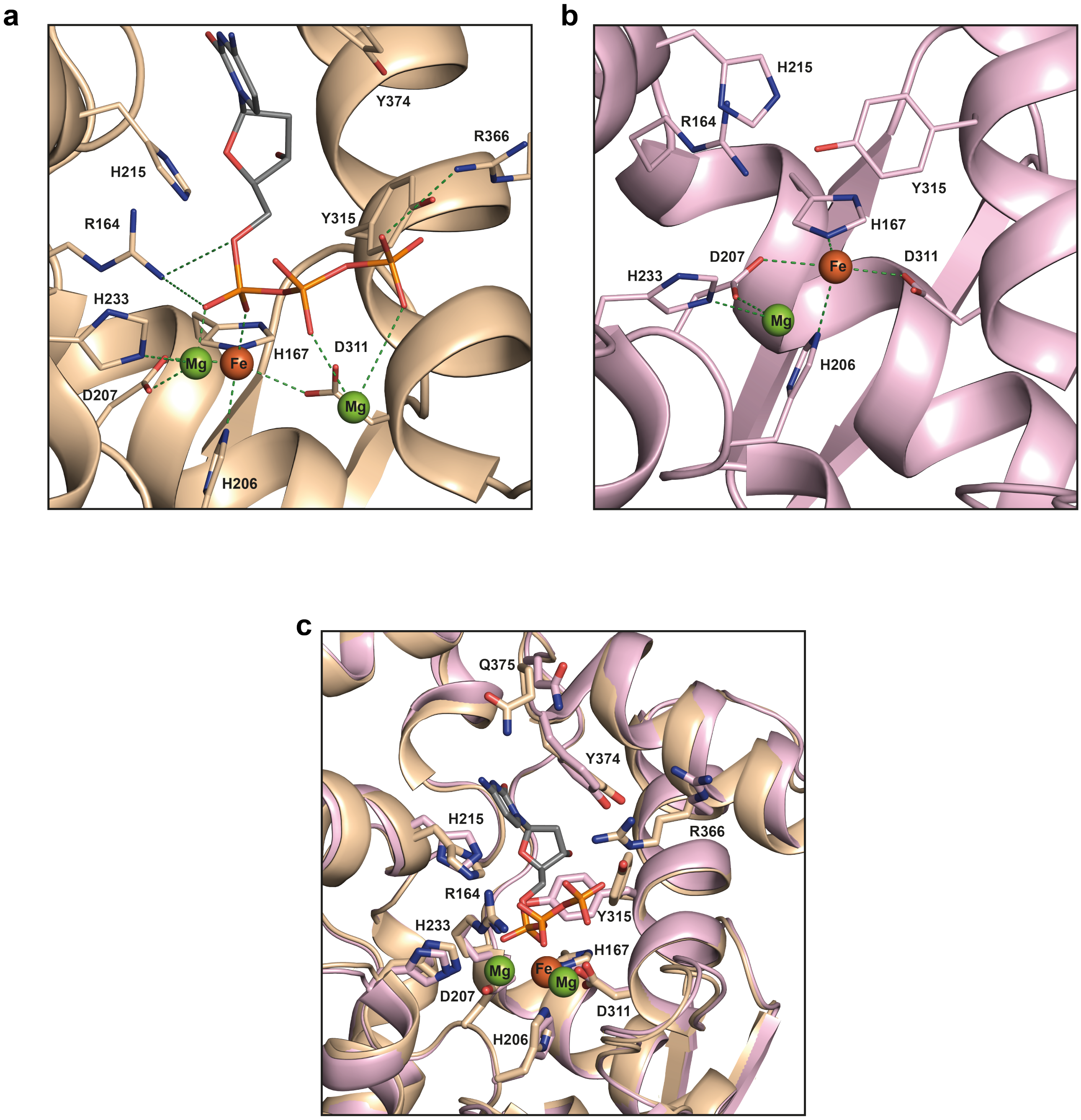

Supplementary Fig. 12. | Active site residues, metal ions and nucleotide co-ordination

Active site of Monomer-A in (**a**) the State-I (bound) and (**b**) the State-III (product released) configuration. The protein backbone is shown in cartoon representation. Active site residues and the bound nucleotide, modelled as dCTP, are shown in stick representation, metal ions are shown as spheres, coloured by atom type. Metal ion ligand interactions and hydrogen bonding are represented by green dashed lines, distances 2 - 3.4 Å. (**c**) Structural overlay of the monomer-A active site in the State-I and State-III structures. The protein backbone is

coloured wheat, State-I and pink State-III. Active site residues and the bound nucleotide, in State-I, are shown in stick representation, metal ions are shown as spheres, coloured by atom type. The side chains of the active site residues H215, Y315, R366, Y374 and Q375 adopt different conformations in the State-I and State-III structures in response to dNTP binding and product release. The conformation of HD residues H167, D206, H207, D311 along with R164 and H233 are invariant between states.

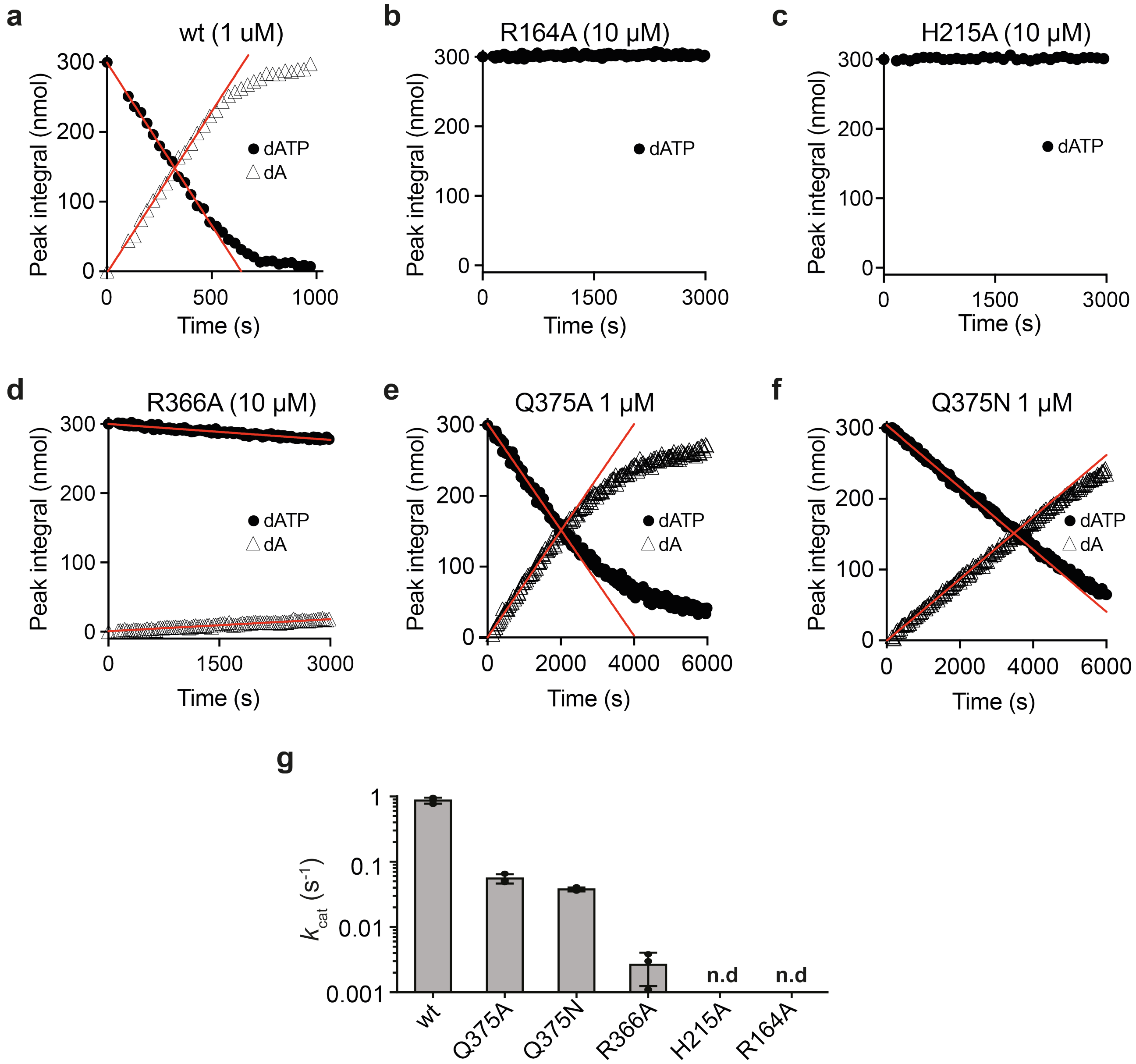

Supplementary Fig. 13. | Catalytic activity of SAMHD1 active site mutants. (a-f) ^{1}H NMR analysis of GTP stimulated dATP hydrolysis by wt-SAMHD1 and active site mutants R164A, H215A, R366A, Q375A and Q375N. ^{1}H NMR data was recorded for SAMHD1 hydrolysis reactions containing wt-SAMHD1, Q375A and Q375N at 1 µM or active site mutants R164A, H215A and R366A at 10 µM with 0.2 mM GTP AL1-activator and 0.5 mM dATP. In each panel, the integral of resolved substrate and product peak resonances is plotted against time. Initial rates of hydrolysis were determined from slopes (red lines) derived from the data measured in the linear part of the reaction. (**g)** Bar chart of the apparent k_{cat} values derived from the data shown in (**a-f**). Points are individual

measurements, bar heights represent the mean and error bars represent s. d. of the mean of three independent measurements, n.d. (not detectable). Source data for **a-g** are provided in the Source Data file.

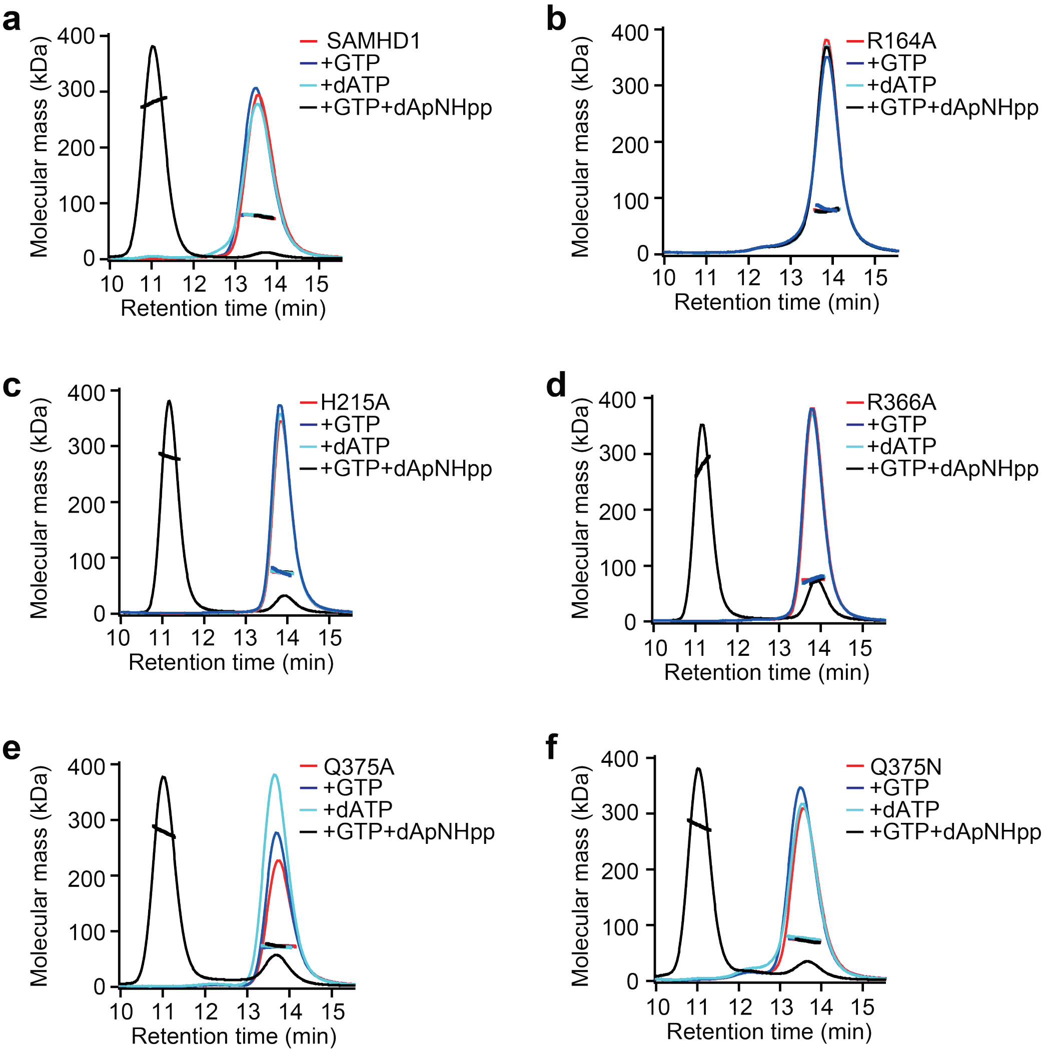

Supplementary Fig. 14. | Tetramerisation of active site mutants. SEC-MALLS analysis of monomer-dimer-tetramer equilibrium of wt-SAMHD1 (**a**) and active site mutants R164A (**b**), H215A (**c**), R366A (**d**), Q375A (**e**) and Q375N (**f**) upon addition of GTP and dApNHpp nucleotides. In each panel, the solid lines are the chromatograms from the output of the differential refractometer and the scatter points are the weight-averaged molar masses determined at 1-second intervals throughout elution of chromatographic peaks. The curves shown are: (red) apo-SAMHD1; (blue) SAMHD1 and 0.2 mM GTP; (cyan) SAMHD1 and 0.5 mM dApNHpp; and (black) SAMHD1, 0.2 mM GTP and 0.5 mM dApNHpp. With the exception of R164A, mutants are catalytically deficient but are competent to induce SAMHD1 tetramerisation.

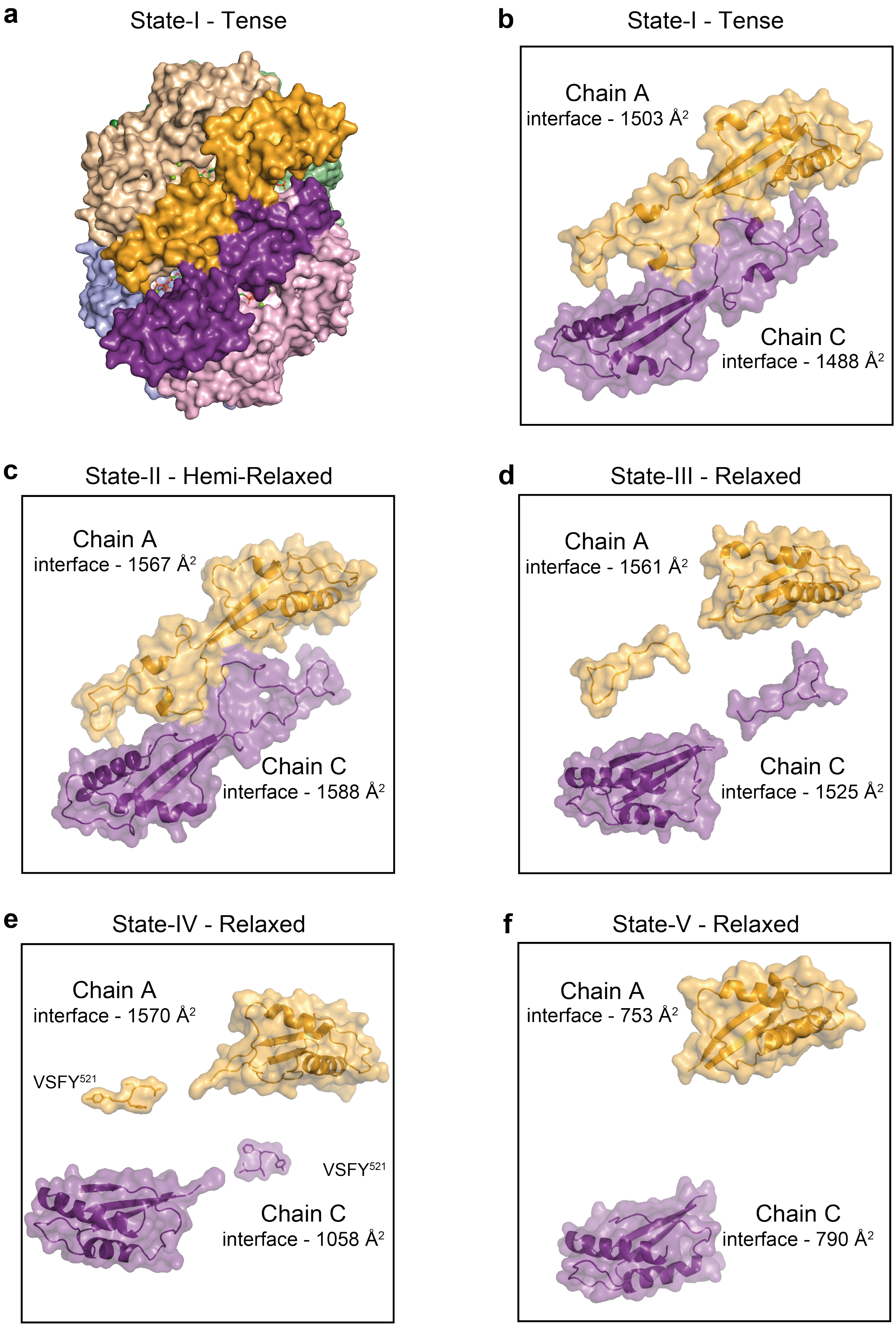

Supplementary Fig. 15. | Regulatory domain order-disorder transition. (a) State-I tetramer shown in surface representation, view onto the Monomer-A/Monomer-C Dimer-2

coloured as in Fig. 1. (**b - f**) The regulatory domains of Monomer-A and Monomer-C are shown for (**a**) State-I, (**b**) State-II, (**c**) State-III, (**d**) State-IV and (**e**) State-V SAMHD1-GTP-dNTP complexes. The view is as in **a**. For each of the states the ordered protein backbone is shown in cartoon and surface representation. The tetrapeptide ^{518}VSFY^{521} from the lobe-linker region that remains ordered in State-IV is shown as sticks. The buried area resulting from the packing of each C-terminal lobe and linker region onto its cognate HD-domain is indicated.

Supplementary Table 1. SAMHD1 Catalytic turnover of combined dNTPs[1]

Substrate	k_{cat} (s^{-1})
dATP	0.131±0.035[2]
dGTP	0.249±0.046
dCTP	0.201±0.040
TTP	0.162±0.030
Total dNTP	0.743±0.076

[1]Data derived from ^{1}H NMR measurements in Supplementary Figure 1. [2]Errors are SD of four independent measurements.

Supplementary Table 2. SAMHD1 Cryo-EM time course data collection

	Inhibitor	[1]T = 0	T = 10	T = 300	T = 600	T = 2400	T = 5400
**Data collection**							
Magnification	130,000	130,000	130,000	75,000	75,000	75,000	75,000
Voltage (kV)	300	300	300	300	300	300	300
Total exposure ($e^-/Å^2$)	48.6	48.6	48.6	33.0	33.0	33.0	33.0
Defocus range (μm)	-1.0 – -3.5	-1.0 – -3.5	-1.0 – -3.5	-1.0 – -3.5	-1.0 – -3.5	-1.0 – -3.5	-1.0 – -3.5
Pixel size (Å)	1.08	1.08	1.08	1.09	1.09	1.09	1.09

[1]reaction time (sec) before freezing for data collection.

Supplementary Table 3a. SAMHD1 map-model correlation, chains[1]

Map	Model	Chain A	Chain B	Chain C	Chain D
I	State-I	0.83	0.83	0.83	0.83
I	State-II	0.71	0.78	0.73	0.79
I	State-III	0.58	0.78	0.57	0.78
I	State-IV	0.44	0.70	0.46	0.71
I	State-V	0.37	0.57	0.37	0.58
**Map**	**Model**	**Chain A**	**Chain B**	**Chain C**	**Chain D**
II	State-I	0.71	0.82	0.70	0.82
II	State-II	0.72	0.81	0.72	0.82
II	State-III	0.70	0.82	0.71	0.82
II	State-IV	0.57	0.75	0.60	0.76
II	State-V	0.50	0.62	0.50	0.63
**Map**	**Model**	**Chain A**	**Chain B**	**Chain C**	**Chain D**
III	State-I	0.57	0.81	0.56	0.81
III	State-II	0.64	0.81	0.63	0.81
III	State-III	0.79	0.84	0.79	0.84
III	State-IV	0.66	0.78	0.68	0.78
III	State-V	0.59	0.67	0.58	0.67
**Map**	**Model**	**Chain A**	**Chain B**	**Chain C**	**Chain D**
IV	State-I	0.46	0.72	0.50	0.72
IV	State-II	0.54	0.75	0.57	0.73
IV	State-III	0.64	0.76	0.67	0.74
IV	State-IV	0.65	0.74	0.66	0.73
IV	State-V	0.59	0.66	0.59	0.66
**Map**	**Model**	**Chain A**	**Chain B**	**Chain C**	**Chain D**
V	State-I	0.45	0.71	0.46	0.71
V	State-II	0.53	0.74	0.52	0.73
V	State-III	0.65	0.75	0.64	0.75
V	State-IV	0.66	0.75	0.67	0.75
V	State-V	0.66	0.72	0.66	0.72

[1]Map-model cross correlation coefficient (calculated in Phenix map-model validation) for placement of Chains A-D from State-I to V models into the State-I to -V maps. Decreasing correlation is shown by darker shading.

Supplementary Table 3b. SAMHD1 map-model correlation, nucleotide sites[2]

Site-dNTP	Model	BAD	ABC	DCB	CDA
AL1-GTP	State-I	0.86	0.86	0.86	0.86
	State-II	0.86	0.82	0.87	0.81
	State-III	0.88	0.79	0.87	0.78
	State-IV	0.78	0.59	0.74	0.66
	State-V	0.72	0.62	0.74	0.63
**AL2- dATP**	**Model**	**BAD**	**ABC**	**DCB**	**CDA**
	State-I	0.88	0.88	0.88	0.88
	State-II	0.85	0.79	0.86	0.78
	State-III	0.88	0.75	0.88	0.74
	State-IV	0.75	0.45	0.74	0.51
	State-V	0.65	0.45	0.68	0.47
**Active -dCTP**	**Model**	**Chain A**	**Chain B**	**Chain C**	**Chain D**
	State-I	0.70	0.70	0.70	0.70
	State-II	0.47	0.72	0.46	0.72
	State-III	0.28	0.73	0.28	0.72
	State-IV	0.28	0.68	0.27	0.64
	State-V	0.27	0.65	0.27	0.67

[2]Map-model cross correlation coefficient (calculated in Phenix map-model validation) for placement of nucleotides from the State-I model into State-I to -V maps. Decreasing correlation is shown by darker shading.

Supplementary Table 4. Nucleotide and metal contents of SAMHD1 structures

	Active site (nucleotide)	Active site (metal)	AL1 (nucleotide)	AL2 (nucleotide)	AL1/2 (Metal)
**State-I**					
Ch-A (AL site ABC)	dCTP	Fe, Mg2, Mg3	GTP	dATP	Mg1
Ch-C (AL site CDA)	dCTP	Fe, Mg2, Mg3	GTP	dATP	Mg1
Ch-B (AL site BAD)	dCTP	Fe, Mg2, Mg3	GTP	dATP	Mg1
Ch-D (AL site DCB)	dCTP	Fe, Mg2, Mg3	GTP	dATP	Mg1
**State-II**					
Ch-A (AL site ABC)	dC + $P_30_{10}H_4$	Fe, Mg3	GTP	dATP	Mg1
Ch-C (AL site CDA)	dC + $P_30_{10}H_4$	Fe, Mg3	GTP	dATP	Mg1
Ch-B (AL site BAD)	dCTP	Fe, Mg2, Mg3	GTP	dATP	Mg1
Ch-D (AL site DCB)	dCTP	Fe, Mg2, Mg3	GTP	dATP	Mg1
**State-III**					
Ch-A (AL site ABC)	/	Fe, Mg3	GTP	dATP	Mg1
Ch-C (AL site CDA)	/	Fe, Mg3	GTP	dATP	Mg1
Ch-B (AL site BAD)	dCTP	Fe, Mg2, Mg3	GTP	dATP	Mg1
Ch-D (AL site DCB)	dCTP	Fe, Mg2, Mg3	GTP	dATP	Mg1
**State-IV**					
Ch-A (AL site ABC)	/	Fe, Mg3	GTP	dATP	Mg1
Ch-C (AL site CDA)	/	Fe, Mg3	GTP	dATP	Mg1
Ch-B (AL site BAD)	dCTP	Fe, Mg3	GTP	dATP	Mg1
Ch-D (AL site DCB)	dCTP	Fe, Mg3	GTP	dATP	Mg1
**State-V**					
Ch-A (AL site ABC)	/	Fe	GTP	/	/
Ch-C (AL site CDA)	/	Fe	GTP	/	/
Ch-B (AL site BAD)	dCTP	Fe	GTP	dATP	Mg1
Ch-D (AL site DCB)	dCTP	Fe	GTP	dATP	Mg1

Supplementary Table 5. Fraction of SAMHD1 states over time of reaction[1]

Time of reaction (sec)	Fraction turnover[2]	Dimer	State-I Tetramer	State-II Tetramer	State-III Tetramer	State-IV Tetramer	State-V Tetramer
T = 0	0.00	1.00	0.00	0.00	0.00	0.00	0.00
T = 10	0.00	0.50	0.15	0.25	0.10	0.00	0.00
T = 300	0.24	0.07	0.23	0.41	0.23	0.06	0.00
T = 600	0.48	0.00	0.25	0.42	0.27	0.06	0.00
T = 2400	0.99	0.00	0.00	0.07	0.52	0.28	0.13
T = 5400	1.00	0.00	0.00	0.00	0.10	0.20	0.70

[1]Derived from particle counts for each state after 3D variability analysis, except Dimer from initial 3D classification; [2]Derived from ^{1}H NMR data in Supplementary Figure 1.

Supplementary Table 6. SAMHD1 catalysis of active site and transducer mutants

Sample	Substrate	AL1	AL2	k_{cat} (s^{-1})	*(f)*[1]
w.t.	dATP	GTP	dATP	0.864±0.092	-
R164A	dATP	GTP	dATP	*n.m*[2]*.*	
H215A	dATP	GTP	dATP	*n.m.*	
R366A	dATP	GTP	dATP	0.0026±0.0014	332
Q375A	dATP	GTP	dATP	0.055±0.009	15.7
Q375N	dATP	GTP	dATP	0.038±0.0026	22.7

[1]Fold reduction in k_{cat}(*w.t.*/mutant); [2]not measurable, below the limit of reliable detection, 0.00075 s^{-1}. Errors are SD of at least three independent measurements.

Supplementary Table 7a. Buried surface area (Å^2) – intrachain regulatory and catalytic domain interface

Chains	State-I	State-II	State-III	State-IV	State-V
A-A	1503	1567	1561	1070	753
C-C	1488	1588	1525	1058	790
Mean (A & C)	1496	1578	1543	1064	772
B-B	1503	1482	1508	1530	1476
D-D	1511	1471	1482	1533	1569
Mean (B & D)	1507	1477	1495	1532	1523

Supplementary Table 7b. Buried surface area (Å^2) – interchain CTD-lobe linker dimer interface

Chains	State-I	State-II	State-III	State-IV	State-V
A-C	724	540	0	0	0
C-A	720	565	0	0	0
Mean (A & C)	722	553	/	/	/
B-D	708	632	617	616	595
D-B	711	624	620	614	608
Mean (B & D)	710	627	619	615	602

Supplementary Table 7c. Buried surface area (Å^2) – interchain interface

Chains	State-I	State-II	State-III	State-IV	State-V
A-B	1695	1711	1678	1714	1754
C-D	1702	1728	1688	1671	1698
A-C	1242	1078	472	424	350
B-D	1236	1131	1174	1181	1099
A-D	339	352	320	303	320
B-C	341	336	358	311	298

Supplementary Table 8. Primers for SAMHD1 mutagenesis

Construct		Primers (5' - 3')[#]
R164A	FWD	GGAGCTTCACACAATGCATTTGAGCATAGTCTACT
	REV	AGTAGACTATGCTCAAATGCATTGTGTGAAGCTCC
H215A	FWD	GTCATGGGCCATTTTCTGCCATGTTTGATGGACGATTTATTC
	REV	GAATAAATCGTCCATCAAACATGGCAGAAAATGGCCCATGAC
R366A	FWD	GACATGTTCCACACTGCCCAACTCTTTACACCG
	REV	CGGTGTAAAGAGTTGGCAGTGTGGAACATGTC
Q375A	FWD	CACCGTAGAGCTTATGCACACAAAGTTGGCAAC
	REV	GTTGCCAACTTTGTGTGCATAAGCTCTACGGTG
Q375N	FWD	CACCGTAGAGCTTATAACCACAAAGTTGGCAAC
	REV	GTTGCCAACTTTGTGGTTATAAGCTCTACGGTG

[#]Mutagenized codons are highlighted and underlined
