# Supplementary material for: Platform-directed allostery and quaternary structure dynamics of SAMHD1 catalysis
Source: Nat Commun. 2024 May 6;15:3775. doi: 10.1038/s41467-024-48237-w (PMC11074143; doi:10.1038/s41467-024-48237-w)
Supplement: Supplementary file 3 — Description of Additional Supplementary Files [file 41467_2024_48237_MOESM3_ESM.pdf]

Description of Additional Supplementary Files

File Name: Supplementary Movie 1

Description: Domain motions from State-I to V.

The SAMHD1 tetramer is shown in cartoon representation and semitransparent surface. the N-terminal catalytic domain, and C-terminal regulatory domains of the four monomers are coloured wheat and orange (Monomer-A), pale green and green (Monomer-B), pink and magenta (Monomer-C), pale blue and blue (Monomer-D). The movie was created in Pymol 2.5.2 and edited in Adobe Premiere Pro 2024. It shows the transition from State-I through State-V and then State-V returning to State-I highlighting the motions of the C-terminal regulatory lobes and linkers in the Monomer-A/MonomerC Dimer-2 interface that transitions from tense to relaxed and their static nature at the Monomer-B/Monomer-D Dimer-2 interface that remains tense.
